# Learning Perfectly Secure Cryptography to Protect Communications with Adversarial Neural Cryptography

**DOI:** 10.3390/s18051306

**Published:** 2018-04-24

**Authors:** Murilo Coutinho, Robson de Oliveira Albuquerque, Fábio Borges, Luis Javier García Villalba, Tai-Hoon Kim

**Affiliations:** 1 Cybersecurity INCT Unit 6, Decision Technologies Laboratory—LATITUDE, Electrical Engineering Department (ENE), Technology College, University of Brasília (UnB), 70.910-900 Brasília-DF, Brazil; murilo.coutinho@redes.unb.br (M.C.); robson@redes.unb.br (R.d.O.A.); 2 National Laboratory for Scientific Computing; 25.651-075 Petrópolis-RJ, Brazil; borges@lncc.br; 3 Group of Analysis, Security and Systems (GASS), Department of Software Engineering and Artificial Intelligence (DISIA), Faculty of Computer Science and Engineering, Office 431, Universidad Complutense de Madrid (UCM), Calle Profesor José García Santesmases, 9, Ciudad Universitaria, 28040 Madrid, Spain; 4 Department of Convergence Security, Sungshin Women’s University, 249-1 Dongseon-Dong 3-ga, Seoul 136-742, Korea; taihoonn@daum.net

**Keywords:** Adversarial Neural Cryptography, Artificial Intelligence, Chosen-Plaintext Attack, Cryptography, Neural Network, One-Time Pad

## Abstract

Researches in Artificial Intelligence (AI) have achieved many important breakthroughs, especially in recent years. In some cases, AI learns alone from scratch and performs human tasks faster and better than humans. With the recent advances in AI, it is natural to wonder whether Artificial Neural Networks will be used to successfully create or break cryptographic algorithms. Bibliographic review shows the main approach to this problem have been addressed throughout complex Neural Networks, but without understanding or proving the security of the generated model. This paper presents an analysis of the security of cryptographic algorithms generated by a new technique called Adversarial Neural Cryptography (ANC). Using the proposed network, we show limitations and directions to improve the current approach of ANC. Training the proposed Artificial Neural Network with the improved model of ANC, we show that artificially intelligent agents can learn the unbreakable One-Time Pad (OTP) algorithm, without human knowledge, to communicate securely through an insecure communication channel. This paper shows in which conditions an AI agent can learn a secure encryption scheme. However, it also shows that, without a stronger adversary, it is more likely to obtain an insecure one.

## 1. Introduction

A significant improvement in Artificial Intelligence (AI) has been achieved. Nowadays, several systems overcame human capabilities in human-like tasks as image recognition [1,2], speech recognition [3], driving cars [4] and playing intuitive games [5]. A natural question to be asked is whether AI will be, someday, better than humans to design or break cryptography.

There are some papers in the literature trying to use machine learning techniques to design new cryptographic algorithms. Most of these works proposed encryption schemes designed with Neural Networks (NN) as a tool to create non-linearity [6,7,8,9]. It could be argued that these proposals are not Artificial Intelligence as there is not an agent learning based on security concepts. Also, in the hands of experienced crypto experts, theses algorithms turned out to be badly broken [10,11,12,13].

More recently [14], Abadi and Andersen suggested a different approach, namely Adversarial Neural Cryptography (ANC), in which three agents, Alice, Bob and Eve, compete in a dispute. Basically, Eve is a NN that tries to eavesdrop on Alice and Bob’s communication. Alice and Bob, which also are NN, try to learn how to protect their communication from Eve. Their idea is different since the agents are learning about security by themselves. However, a possible critic to their work is that they used complex Convolutional Neural Networks and did not show what cryptosystem their system had learned. Naturally, being secure against another neural network means nothing in terms of real security.

In this work, we analyze the algorithms generated by the ANC technique to understand its security. To do this, we designed a small NN capable of generalizing binary operations to a continuum space allowing the back-propagation algorithm to work properly. We designed this NN in a way that would be possible, but not necessary, to learn the One-Time Pad (OTP), which is well-known for being information-theoretically secure [15]. In other words, the OTP is unbreakable under some assumptions. Using this neural network, we show that the ANC model is not good enough to generate secure cryptosystems even using a simple NN.

To overcome these limitations, we propose an improvement to the ANC methodology using the concept of the Chosen-Plaintext Attack (CPA) [16], leading to what we called CPA-ANC. Since our NN is very simple, we were capable of reasoning when the learned model was in fact secure and why. With the proposed CPA-ANC methodology our NN learned the OTP, a secure cryptosystem.

The main contribution of this work is to demonstrate that an Artificial-Intelligent agent can learn a secure encryption algorithm without human knowledge. Previous work had similar contribution for games [5]. However, the conditions to achieve a secure algorithm are difficult to obtain and being more likely to get an insecure encryption algorithm if the agent is not in a well-crafted environment.

This paper is organized as follows: in Section 2 we presented related work. With more detail in Section 2.1 we review Neural Cryptography and in Section 2.2, we present the ANC methodology of [14].

In Section 3 we present our main contributions. In particular, in Section 3.1 we propose an improvement to the ANC methodology called CPA-ANC which uses the Chosen-Plaintext Attack to improve the security of the algorithms that the agents can learn. Also, in Section 3.2 we proposed a simple NN capable of learning the One-Time Pad.

In Section 4 we test the proposed methodology against the traditional ANC. Specifically, in Section 4.2 we trained the proposed NN without an adversary showing the Alice and Bob usually will communicate properly but without any form of encryption. In Section 4.3 we trained the proposed NN under the ANC methodology showing Alice and Bob can protect their communication from Eve but with an insecure encryption scheme. In Section 4.4, we train the proposed NN under the CPA-ANC methodology leading Alice and Bob to protect their communication from Eve generating a secure encryption scheme with very high probability, namely the OTP.

In Section 5, we show a concise comparison with related work and, finally, in Section 6 we present the conclusions, open questions and directions to future research.

## 2. Related Work

In this section, we review some works that used Neural Networks in Cryptography. More importantly, we explain how Adversarial Neural Cryptography works as proposed in [14].

### 2.1. Neural Cryptography

In 2002, Kanter et al. [6] proposed a new key exchange protocol between two parties using the notion of chaotic synchronization, which makes it possible for two weakly interacting chaotic systems to converge even though each one of them continues to move in a chaotic way. In Kanter’s protocol, each party has a NN that starts in a random state and at each round it updates itself and then reveals one bit of information about its state to the other party. They also show in [6] that an attacker who uses an identical neural network with the same learning procedure is extremely unlikely to synchronize his network with the other parties. In the same year, however, Shamir et al. [10] broke the system using three different techniques.

Afterwards, several papers used chaotic NN to propose encryption and hash algorithms [7,8,9]. All these papers used the chaotic NN as a tool for randomness only, then we do not consider these works as AI. Nevertheless, all these algorithms were also broken [11,12,13].

NNs were also used to develop pseudo-random number generators (PRNG) [17,18,19]. In these works the parameters of the NNs were considered the seed of the PRNG and the randomness were tested with tools like the NIST random number generator test suit.

### 2.2. Adversarial Neural Cryptography

A recent work defined the concept of ANC [14]. Abadi and Andersen proposed a system in which 2 NNs named Alice and Bob tried to exchange a message while limiting what a third NN, named Eve, could learn from eavesdropping the communication.

In their work, they did not prescribe specific cryptographic algorithms to these neural networks. In fact, they showed that Alice and Bob could learn how to perform forms of encryption and decryption successfully denying Eve any information about the message. However, in their work, they did not show any solution learned by the networks. In this section, we describe their system to give a general understanding of the mechanics of ANC.

#### 2.2.1. System Organization

**Figure 1:**
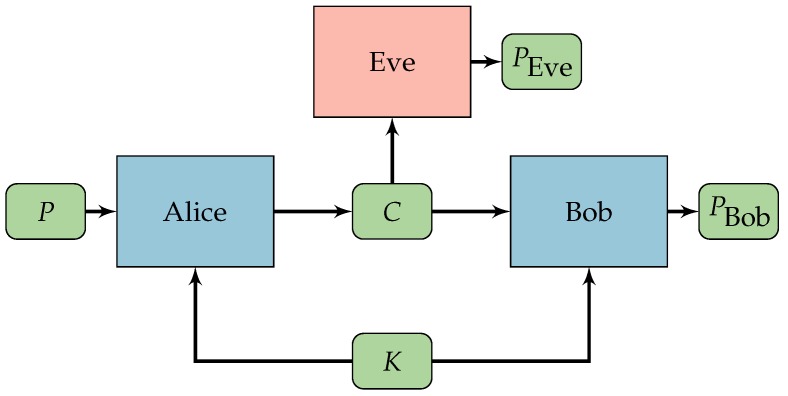
Alice, Bob, and Eve, with a symmetric cryptosystem.

The system of ANC is designed with the classical cryptographic scenario involving three parties (Alice, Bob and Eve) showed in Figure 1. Alice and Bob share a secret key *K* and wish to communicate the plaintext *P* securely. Eve is a passive attacker that wishes to eavesdrop on their communications trying to gain information about *P* through the ciphertext *C*.

In the ANC model, Alice, Bob, and Eve are all neural networks. As is common for neural networks, Alice, Bob, and Eve work over tuples of floating-point numbers, rather than sequences of bits. In other words, *K*, *P*, $P_{\mathrm{Bob}}$, $P_{\mathrm{Eve}}$, and *C* are all tuples of floating-point numbers.

#### 2.2.2. Methodology

As neural networks, Alice, Bob and Eve have parameters that will be denoted by $\theta_{A}$, $\theta_{B}$ and $\theta_{E}$, respectively. Also, let’s define $E_{A}\left(\theta_{A},P,K\right)$ as Alice’s output (maybe an encryption) on inputs *P* and *K*, $D_{B}\left(\theta_{B},C,K\right)$ as Bob’s output (decryption) on inputs *C* and *K*, and $D_{E}\left(\theta_{E},C\right)$ as Eve’s output on input *C*.

To measure the distance between the true plaintext and an estimated value, the L1 distance is used, the L1 distance is defined by:

(1) $$d\left(P,P^{\prime }\right)=\frac{1}{N}\sum_{i=1}^{N}\left|p_{i}-p_{i}^{\prime }\right|,$$

where *N* is the length of plaintexts.

Eve’s goal is simple: to reconstruct *P* accurately. To achieve this goal, Eve’s loss function per-example is defined by

(2) $$L_{E}\left(\theta_{A},\theta_{E},P,K\right)=d\left(P,D_{E}\left(\theta_{E},E_{A}\left(\theta_{A},P,K\right)\right)\right).$$

Intuitively, $L_{E}\left(\theta_{A},\theta_{E},P,K\right)$ represents how much Eve is wrong when the plaintext is *P* and the key is *K*. The loss function for Eve over the distribution of plaintexts and keys is defined by taking an expected value:

(3) $$L_{E}\left(\theta_{A},\theta_{E}\right)=E_{P,K}\left[L_{E}\left(\theta_{A},\theta_{E},P,K\right)\right]$$

Thus, one can find the “optimal Eve” by minimizing the loss:

(4) $$O_{E}\left(\theta_{A}\right)={\mathrm{argmin}}_{\theta_{E}}\left(L_{E}\left(\theta_{A},\theta_{E}\right)\right).$$

Similarly, one can define the per-example loss for Bob and extend it to the distribution on plaintexts and keys:

(5) $$L_{B}\left(\theta_{A},\theta_{B},P,K\right)=d\left(P,D_{B}\left(\theta_{B},E_{A}\left(\theta_{A},P,K\right),K\right)\right)$$

(6) $$L_{B}\left(\theta_{A},\theta_{B}\right)=E_{P,K}\left[L_{B}\left(\theta_{A},\theta_{B},P,K\right)\right].$$

Alice and Bob want to communicate clearly, but they also want to hide their communication from Eve. Thus, it is useful to define a loss function for Alice and Bob by combining $L_{B}$ and the optimal value of $L_{E}$:

(7) $$L_{AB}\left(\theta_{A},\theta_{B}\right)=L_{B}\left(\theta_{A},\theta_{B}\right)-L_{E}\left(\theta_{A},O_{E}\left(\theta_{A}\right)\right).$$

Finally, one can obtain the “optimal Alice and Bob” by minimizing $L_{AB}\left(\theta_{A},\theta_{B}\right)$:

(8) $$\left(O_{A},O_{B}\right)={\mathrm{argmin}}_{\left(\theta_{A},\theta_{B}\right)}\left(L_{AB}\left(\theta_{A},\theta_{B}\right)\right).$$

#### 2.2.3. Training

ANC is an unsupervised learning technique. Training begins with all NN (Alice, Bob and Eve) initialized randomly and without any data. For random examples of plaintexts *P*, Alice and Bob try to learn to communicate minimizing the error between the original plaintext *P* and the output from Bob’s network $P_{\mathrm{Bob}}$ and, at the same time, making Eve guesses as random as possible. Afterwards, fixing Alice and Bob networks, Eve tries to decrypt *C* without knowing the key *K*. This process is repeated thousands of times until, hopefully, Alice and Bob find a way to communicate without giving Eve any information.

When training Alice and Bob, one should not attempt to maximize Eve’s reconstruction error. If Eve was completely wrong, then Eve could be completely right in the next iteration by simply flipping all output bits. A more realistic and useful goal for Alice and Bob is to make Eve produce answers indistinguishable from a random guess.

For more details on the ANC technique, please refer to the original paper [14].

## 3. Improvement to the ANC Methodology

In this section, we propose an improvement to the ANC methodology using the Chosen-Plaintext Attack (CPA) which we call CPA-ANC. Additionally, we present a simple NN capable of learning the One-Time Pad which will be used to test this new methodology against the traditional ANC.

### 3.1. Chosen-Plaintext Attack Adversarial Neural Cryptography

As we will see in the experiments of Section 4, the problem with the approach proposed in the original ANC work [14] is that Eve’s job is too hard. It must decrypt a random message having access only to the ciphertext. The consequence is that, under this methodology, Alice and Bob learn to communicate with cryptosystems that are not truly secure. Therefore, one can conclude that Alice and Bob do not have to do much effort to protect themselves against Eve, leading in insecure cryptosystems.

It is possible to improve ANC considering a more robust model of security for Alice, Bob and Eve. Namely, we will let Eve to mount a CPA. Therefore, to be protected against Eve, Alice and Bob will have to find a system secure against the CPA.

**Figure 2:**
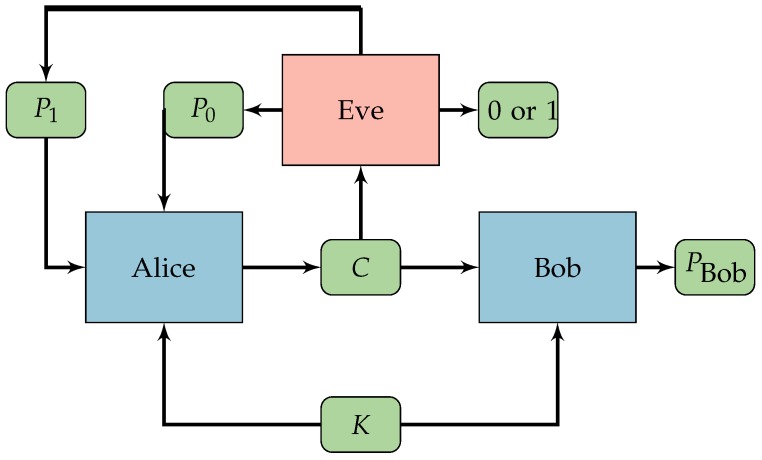
Alice, Bob, and Eve, and the CPA-ANC setup. Alice and Bob share a secret key *K*. Eve chooses two messages $P_{0}$ and $P_{1}$. Alice randomly chooses one message to encrypt producing the ciphertext *C*. Bob uses the key *K* to decrypt *C* producing $P_{\mathrm{Bob}}$. Eve receives the ciphertext *C* and tries to guess which message was encrypted outputting 0 if believes $P_{0}$ was encrypted and 1 if believes $P_{1}$ was encrypted.

In this new setup Eve will choose two messages $P_{0}$ and $P_{1}$ and send them to Alice. Alice will choose one of these messages randomly, encrypt it with the NN obtaining the ciphertext *C* and send it to Eve and Bob. As usual, Bob will decrypt the message with a NN. However, Eve will not try to decrypt *C*, but will only output 0 if it believes $P_{0}$ was encrypted or 1 if it believes $P_{1}$ was encrypted. We call this the CPA-ANC setup and it is illustrated in Figure 2.

In this scenario of CPA-ANC, Alice and Bob will have to find a much better cryptosystem to communicate securely. In Section 4, we will show that this approach can really improve the quality of the solution and that a NN can, in fact, produce secure cryptosystems.

### 3.2. A Simple Neural Network Capable of Learning the One-Time Pad

In this section, we propose a NN complex enough to be able to learn some form of cryptography but simple enough to allow us to reason about its security. To do this, we used a continuous generalization for the operator XOR, which is a well-known binary and non-differentiable operation that happens to be used a lot in cryptography.

Thus, if we want a NN that can perform the XOR operation internally, we need a generalization of the operation. It is possible to generalize the XOR operation using the unit circle by mapping the bit 0 to the angle 0 and the bit 1 to the angle π. In this way, the XOR is equivalent to the sum of the angles.

Note, however, that the sum is a continuous operation. Thus, it is possible to work with angles different from 0 or π, generalizing bits to a continuous space. The following equation defines the mapping of a bit *b* into an angle:

(9) f(b)=arccos(1−2b).

The inverse of *f* provides the mapping of an angle *a* to a “continuous bit”:

(10) $$f^{-1}\left(a\right)=\frac{1-\cos(a)}{2}.$$

**Figure 3:**
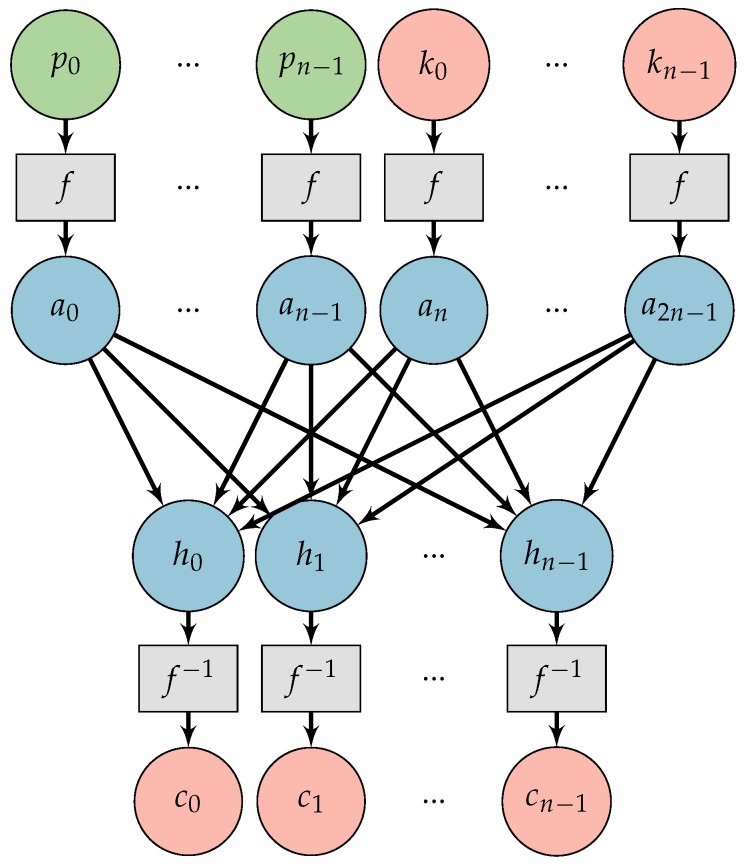
The proposed neural network (*CryptoNet*). The bits of the plaintext are represented by [$p_{0}$, $p_{1}$, …, $p_{n-1}$]. The bits of the key are represented by [$k_{0}$, $k_{1}$, …, $k_{n-1}$]. The function *f* (see Equation (9)) transforms the bits into angles [$a_{0}$, $a_{1}$, … $a_{2n-1}$]. A fully connected layer combines the angles forming the variables [$h_{0}$, $h_{1}$, …, $h_{n-1}$]. The function $f^{-1}$ (see Equation (10)) transforms the combined angles into continuous bits (real numbers in the interval [0,1]) [$c_{0}$, $c_{1}$, …, $c_{n-1}$] representing the ciphertext.

With this operator, we can introduce a small NN in Figure 3 for learning an OTP. We will refer to this NN as *CryptoNet*.

Basically, *CryptoNet* receives as input the plaintext and the key and, for each bit received, applies the transformation defined in Equation (9), resulting in angles. The next step is a standard matrix multiplication followed by the inverse transformation defined in Equation (10) resulting in the ciphertext. Note that the ciphertext is not composed of bits but by floating number between 0 and 1.

Mathematically, the fully connected layer of *CryptoNet*, is doing the following operation:

(11) $${\left(\begin{array}{c} h_{0}\\ h_{1}\\ \vdots \\ h_{n-1} \end{array}\right)}^{⊤}={\left(\begin{array}{c} a_{0}\\ \vdots \\ a_{n-1}\\ a_{n}\\ \vdots \\ a_{2n-1} \end{array}\right)}^{⊤}W_{2n,n},$$

where $W_{2n,n}$ is a matrix of weights with 2n rows and *n* columns, $a_{0},\ldots ,a_{2n-1}$ are angles obtained from the plaintext and the key, and $h_{0},\ldots ,h_{n-1}$ are hidden variables.

Through the remainder of the paper, we will denote the *CryptoNet* mathematically as the function

(12) $$C=\zeta_{n}\left(W,P,K\right),$$

where W is the matrix of weights defined above and P,K,C are vectors of *n* bits of input, key and output, respectively.

The *CryptoNet* can learn to combine the input in many ways. Since the input bits are mapped to the angles 0 or π, if all the weights (elements of $W_{2n,n}$) of the connections are integers, the result would be equivalent to XOR operations of the input bits.

Therefore, it is possible to learn an OTP with this network. It should be noted, however, that it is very unlikely to find an OTP randomizing the weights as real numbers since the probability of such event tends to 0.

With this model, we can train the network using ANC and reason about the results. Our goal is to understand if a NN can learn an OTP by itself. Moreover, we want to study and define conditions on the ANC model that can lead to a secure cryptosystem consistently.

## 4. Results

In this section, we test whether the proposed NN can learn a secure encryption system under three different scenarios. First, we train Alice and Bob to communicate without any adversary, showing that they will learn to communicate properly but without any encryption scheme. Then, we train Alice and Bob to communicate under the ANC setup showing that they will learn to communicate with a weak encryption scheme. Finally, we train Alice and Bob under the proposed CPA-ANC setup showing that they learn to communicate securely with high probability using the One-Time Pad.

### 4.1. Method

In all our tests, we trained two agents, Alice and Bob, using the same *CryptoNet*. The goal for Alice and Bob, in this case, was to learn to communicate using a single network. Thus, suppose Alice use an encryption function E(B,K) and Bob use a decryption function D(B,K), we defined these functions as a single *CryptoNet*:

(13) $$D\left(B,K\right)=E\left(B,K\right)=\zeta_{n}\left(W,B,K\right)$$

Thus, Alice and Bob must work together to find a single *CryptoNet* such that its inverse is itself. Therefore, to communicate properly, it should be the case that

(14) $$\zeta_{n}\left(W,\zeta_{n}\left(W,P,K\right),K\right)=P,$$

where *P* is the plaintext and *K* is the key.

In our tests, we initialized the weight matrix W randomly. The training, as in [14], relies on estimated values calculated over “minibatches” of hundreds or thousands of examples alternating the training of Eve with that of Alice and Bob. Since this process is intensive and time consuming for large keys, we trained the model with small plaintexts and keys: 4-bit (n=4), 8-bit (n=8) and 16-bit (n=4).

Unlike the standard training process of NN, here we do not have a clear concept of convergence. This is because when Eve changes its network, then Alice and Bob objective function also change as consequence. Therefore, we used two stopping criteria:

1. If the decryption error of Bob is very close to zero and Eve’s attack are as bad as random guesses, then we stop. In this case, we say we had convergence or a success.
2. If the first stop criterion is not reached in 100.000 rounds, we stop. Here a round is completed when Alice and Bob are trained and then Eve is trained. If this happens we say we did not have convergence or a failure.

We trained this model using *Tensorflow* [20], a machine learning framework in python. Also, we used *Adam* optimizer [21] with a learning rate of 0.0002. We chose the learning rate and other hyperparameters adjusting the values empirically to avoid overfitting and underfitting as explained in the textbook [22]., which was defined empirically.

To obtain a meaningful solution in terms of XOR operations, we define Algorithm 1:

**Algorithm 1:** Testing a discrete *CryptoNet*
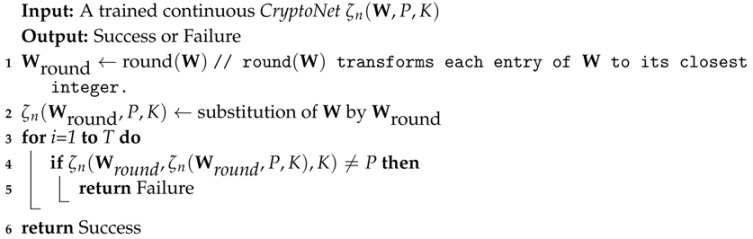


### 4.2. Training without an Adversary

Our first approach was to train two agents, Alice and Bob, to communicate without an adversary. In other words, we want Alice to communicate with Bob without considering Eve. Note that, in this case, we are not using the ANC technique, just a straight forward communication.

In this case, being *M* random examples of plaintexts $\left[P^{\left(0\right)},P^{\left(1\right)},\ldots ,P^{\left(M-1\right)}\right]$ and keys $\left[K^{\left(0\right)},K^{\left(1\right)},\ldots ,K^{\left(M-1\right)}\right]$, we trained *CryptoNet* by minimizing the loss function

(15) $$L_{AB}=\frac{1}{M}\sum_{i=0}^{M-1}d\left(P^{\left(i\right)},\zeta_{n}\left(W,\zeta_{n}\left(W,P^{\left(i\right)},K^{\left(i\right)}\right),K^{\left(i\right)}\right)\right)$$

where the function *d* is the L1 distance defined by

(16) $$d\left(P,P^{\prime }\right)=\frac{1}{N}\sum_{i=1}^{N}\left|p_{i}-p_{i}^{\prime }\right|.$$

We tested several models using Algorithm 1, the results are presented in Table 1. All the trials were successful since Alice and Bob were able to communicate without errors. However, all the trials resulted in an insecure algorithm. As an example, the following algorithm was found in one of the trials:

(17) $$\zeta_{n}\left(W_{\mathrm{round}},P,K\right)={\left[\begin{array}{c} p_{1}\\ p_{0}\\ p_{6}\\ p_{3}\\ p_{4}\\ p_{5}\\ p_{2}\\ p_{7} \end{array}\right]}^{⊤},$$

where $P=[p_{0},p_{1},\ldots ,p_{7}]$ and $K=[k_{0},k_{1},\ldots ,k_{7}]$. Clearly, this solution respects Equation (14).

Note that the key was not used in the learned algorithm of Equation (17). None of the trials resulted in a secure cryptosystem. Naturally, as Alice and Bob were trained to learn to communicate through a *CryptoNet*, without any safety concerns, there were no expectations that the resulting function would have been secure. In the next section, we will introduce *CryptoNet* in the context of ANC to understand if Eve can help Alice and Bob to define a secure system to communicate.

### 4.3. Training the network with Adversarial Neural Cryptography

In Section 4.2, we demonstrated that Alice and Bob could not find a secure network to communicate without any notion of what security means. One way to give Alice and Bob this kind of notion is to introduce an adversary, named Eve, who wants to gain knowledge about the communication over the channel. To do this we use the ANC model.

We will not define Eve explicitly here because it can be any sufficiently complex NN (for example, the one proposed in [14]), as we will show in the results. Alice and Bob will communicate through a single *CryptoNet*, see Equation (13). Eve will try to learn a NN in which receives as input the ciphertext *C* and outputs the plaintext *P*:

(18) $$P=D_{E}\left(\theta ,C\right),$$

where θ are the learned parameters of the NN.

It follows that given *M* examples of plaintexts $\left[P^{\left(0\right)},P^{\left(1\right)},\ldots ,P^{\left(M-1\right)}\right]$ and ciphertexts $\left[C^{\left(0\right)},C^{\left(1\right)},\ldots ,C^{\left(M-1\right)}\right]$, Eve can learn by minimizing the loss

(19) $$L_{E}=\frac{1}{M}\sum_{i=0}^{M-1}d\left(P^{\left(i\right)},D_{E}\left(\theta ,C^{\left(i\right)}\right)\right),$$

where *d* is given in Equation (16).

Note that in the ANC method, Eve uses the plaintext as a measure of performance when training the NN. However, Eve’s NN does not receive the plaintext as input. Therefore, Eve is applying a ciphertext-only attack.

Alice and Bob want to minimize Eve’s knowledge about the plaintext *P*. In practice, Alice and Bob will learn by minimizing the loss

(20) $$L=L_{AB}-\gamma \min\left(L_{E},0.5\right)$$

where $L_{AB}$ is given in Equation (15), $L_{E}$ is given in Equation (19) and γ is a hyperparameter. The minimum function is used to guarantee that Alice and Bob would not try to maximize Eve’s error, since, in this case, Eve could just flip all bits in the next round, achieving a correct guess. If Eve is just guessing the plaintext, it would be expected to get about half of the bits right, resulting in an average error of 0.5.

As in Section 4.2, we train the networks with *M* random examples of plaintexts $\left[P^{\left(0\right)},P^{\left(1\right)},\ldots ,P^{\left(M-1\right)}\right]$ and keys $\left[K^{\left(0\right)},K^{\left(1\right)},\ldots ,K^{\left(M-1\right)}\right]$. In our tests, we used a “minibatch” of M=128 entries. We used γ=5 in Equation (20) (intuitively this means that is more important to keep Eve’s error high than to reduce Bob’s decryption error). Also, we used the L2 regularization with α=0.1 (4-bits), α=0.05 (8-bits) and α=0.01 (16-bits) (see [22]). All these hyperparameters were determined empirically.

**Figure 4:**
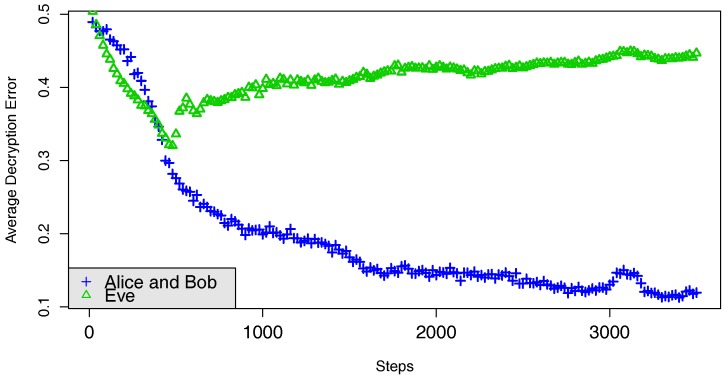
The learning curves forThe challenge between Alice and Bob against Eve who tries to minimize its decryption error. Alice and Bob try to minimize Bob’s decryption error while maximizing Eve’s decryption error. Eve is represented in green and Alice and Bob are represented in blue. The number of steps denote the number of “minibatches” on training phase.

Training alternates between five minibatches for Alice and Bob and 10 minibatches for Eve. The purpose of this ratio is to give a computational edge to Eve without training it so much that it becomes excessively specific to the exact current parameters of Alice and Bob. One example of this process can be seen in Figure 4.

After the training was completed, we used Algorithm 1 to test the learned *CryptoNet*. Unlike in [14], we did not trained Eve again when the training of Alice and Bob was finished. This is because the *CryptoNet* obtained is simple enough, so we can easily reason about its security without the aid of a NN.

Table 2 shows a summary of the results. For each trial, the learned model was considered successful if Alice and Bob could communicate without errors when executing Algorithm 1. A learned *CryptoNet* was considered secure if it learned the OTP. For example, the following *CryptoNet* was learned by Alice and Bob:

(21) $$\zeta_{n}\left(W_{\mathrm{round}},P,K\right)={\left[\begin{array}{c} p_{0}⊕k_{3}\\ p_{3}⊕k_{0}\\ p_{2}⊕k_{0}⊕k_{3}⊕k_{4}⊕k_{5}⊕k_{6}⊕k_{7}\\ p_{1}⊕k_{0}\\ p_{4}⊕k_{1}\\ p_{5}⊕k_{2}\\ p_{6}⊕k_{2}\\ p_{7}⊕k_{1} \end{array}\right]}^{⊤}.$$

This *CryptoNet* was considered a success since Alice and Bob can communicate without errors. Naturally, this encryption in not secure since several plaintext bits were encrypted with the same key bit. However, this *CryptoNet* is secure against Eve. Obviously, any adversary with Eve’s knowledge (any human or NN) cannot recover the plaintext since the plaintext itself was generated randomly. For example, suppose that $c_{1}=c_{3}=0$, then Eve could learn that $c_{1}⊕c_{3}=p_{1}⊕p_{3}$ and infer that $p_{1}=p_{3}$. However, since the plaintext is random, the best Eve could do is to guess $p_{1}$ and $p_{3}$ with the same value but, with this strategy, Eve would get both bits right in 50% of the time and get both bits wrong 50% of the time. Thus, Eve’s output is not better than random, on average.

This demonstrates that the standard ANC technique proposed in [14] is not good enough because it usually does not lead to a secure cryptosystem even using a very simple NN designed to allow a perfectly secure solution. In the next section, we will use the proposed CPA-ANC giving Eve a lot more power forcing Alice and Bob to a better cryptosystem.

### 4.4. Learning the One-Time Pad

To test the proposed CPA-ANC setup, as in previous sections, we considered Alice and Bob sharing a single *CryptoNet* (presented in Figure 3) to encrypt and decrypt. On the other hand, Eve’s network changed a lot. Now Eve is a classifier, receiving $P_{0}$, $P_{1}$ and *C* as input and classifying *C* as 0 if it believes *C* was derived from $P_{0}$ and 1 if it believes *C* was derived from $P_{1}$ .

**Figure 5:**
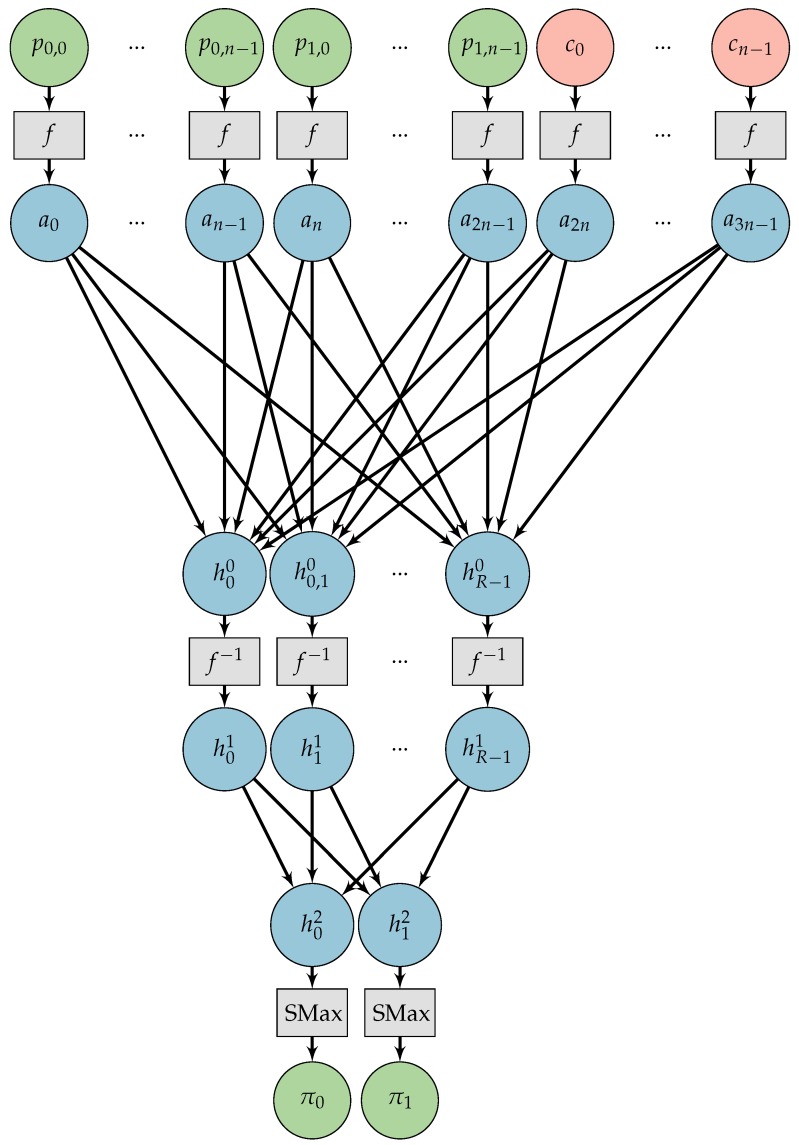
Eve’s neural network (*CPA-CryptoNet*). Eve receives as input two plaintexts $P_{0}$ and $P_{1}$. The bits of each plaintext are represented by [$p_{0,0}$, …, $p_{0,n-1}$] for $P_{0}$ and [$p_{1,0}$, …, $p_{1,n-1}$] for $P_{1}$. Eve also receives the ciphertext *C* represented by [$c_{0}$, …, $c_{n-1}$]. The function *f* (see Equation (9)) transforms the bits into angles [$a_{0}$, $a_{1}$, … $a_{3n-1}$]. A fully connected layer combines the angles generating the hidden variables [$h_{0}^{0}$, $h_{1}^{0}$, …, $h_{R-1}^{0}$], where *R* is the number of rules. The function $f^{-1}$ (see Equation (10)) transforms the combined angles into continuous bits (real numbers in the interval [0,1]) [$h_{0}^{1}$, $h_{1}^{1}$, …, $h_{R-1}^{1}$]. Another fully connected layer brings the hidden variables to logits through a softmax layer resulting in a probability $\pi_{0}$ of *C* being a ciphertext of $P_{0}$ and a probability $\pi_{1}$ of *C* being a ciphertext of $P_{1}$.

We designed Eve with a similar structure as the *CryptoNet*. It receives two plaintexts $P_{0}$ and $P_{1}$, the ciphertext *C*, and transforms the bits into angles through Equation (9). A fully connected layer, combines the angles into a total of *R* rules. Each rule is composed by generalized XOR operations and are transformed back into continuous bits through Equation (10). A second fully connected layer combines the results into logits going through a softmax layer resulting in a probability $\pi_{0}$ of *C* being a ciphertext of $P_{0}$ and a probability $\pi_{1}$ of *C* being a ciphertext of $P_{1}$. Finally, the network outputs 0 if $\pi_{0}>\pi_{1}$ or outputs 1 otherwise. We call this network *CPA-CryptoNet* and it is represented in Figure 5.

We must change the optimization problem to adapt to this new CPA scenario. To do this, given *M* examples of plaintexts $\left[P_{0}^{\left(0\right)},P_{0}^{\left(1\right)},\ldots ,P_{0}^{\left(M-1\right)}\right]$, $\left[P_{1}^{\left(0\right)},P_{1}^{\left(1\right)},\ldots ,P_{1}^{\left(M-1\right)}\right]$ and ciphertexts $\left[C^{\left(0\right)},C^{\left(1\right)},\ldots ,C^{\left(M-1\right)}\right]$, we define the loss for Eve as the cross-entropy:

(22) $$L_{E}=-\frac{1}{M}\sum_{i=0}^{M-1}\sum_{j=0}^{1}t_{j}^{\left(i\right)}\log\left(\pi_{j}^{\left(i\right)}\right)$$

where $t_{j}^{\left(i\right)}=1$ if $C^{\left(i\right)}$ is the ciphertext of $P_{j}^{\left(i\right)}$, and $t_{j}^{\left(i\right)}=0$ otherwise. Thus, Eve learns by minimizing $L_{E}$ while Alice and Bob learn by minimizing *L* given by

(23) $$L=L_{AB}-\gamma \min\left(Err,0.5\right)$$

where $L_{AB}$ is given in Equation (15), Err is Eve’s classification error and γ is a hyperparameter.

To train the networks, we used a “minibatch” of M=128 entries. We used γ=7 in Equation (23). Also, we used the L2 regularization (see [22]) with α=0.1 for 4-bit (n=4) and 8-bit (n=8) key lengths and α=0.015 for 16-bit (n=16) key length. Also, for Eve’s network, we defined the number of rules *R* (see Figure 5) as R=4×n. The number of rules defines the number of linear combinations that Eve can analyze together to attack. Eve should need more equations as the key size grows. Therefore, we set it proportionally to the number of bits of the key. We grew the number of linear combinations until reaching a value in which Eve had great power to break the kind of cryptograms learned by Alice and Bob through the Cryptonet reaching the value of 4×n rules.We chose all these parameters empirically.

**Figure 6:**
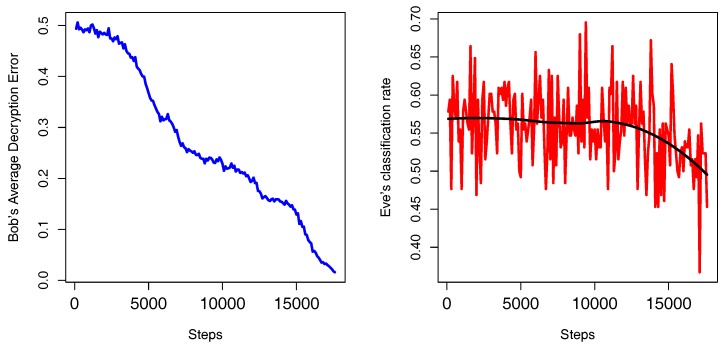
The challenge of Alice and Bob against Eve. Eve tries to maximize its classification rate while Alice and Bob try to minimize Eve’s classification rate and minimize Bob’s decryption error. In the figure on the left, Bob’s decryption error decreases over time in blue color. Also, on the right, one can see that Eve increases its classification rate in red color, however, when Alice and Bob learn a secure cryptosystem, in this case the OTP, Eve’s classification rate becomes no better than random. The number of steps denote the number of “minibatches” on training phase. In black, we have a smooth version of the red curve.

Training alternates between the NN of Alice and Bob and the NN of Eve, with Alice and Bob training for 3 “minibatches”, and then Eve training for 60 “minibatches”. The purpose of this ratio is to give a computational advantage to Eve. One example of this process can be seen in Figure 6.

Table 3 shows a summary of the results. For each trial, the learned model was considered successful if Alice and Bob could communicate without errors when executing Algorithm 1. The model was considered a failure otherwise. A learned *CryptoNet* was considered secure against Eve, if Eve could not extract any information from the ciphertext. For example, the following crypto systems were learned by Alice and Bob:

(24) $$\zeta_{n}\left(W_{\mathrm{round}},P,K\right)={\left[\begin{array}{c} p_{0}⊕k_{5}\\ p_{1}⊕k_{7}\\ p_{2}⊕k_{1}\\ p_{3}⊕k_{0}\\ p_{4}⊕k_{2}\\ p_{5}⊕k_{6}\\ p_{6}⊕k_{3}\\ p_{7}⊕k_{4} \end{array}\right]}^{⊤}\;\mathrm{and}\;\zeta_{n}\left(W_{\mathrm{round}},P,K\right)={\left[\begin{array}{c} p_{0}⊕k_{3}\\ p_{1}⊕k_{10}\\ p_{2}⊕k_{14}\\ p_{3}⊕k_{9}\\ p_{4}⊕k_{5}\\ p_{5}⊕k_{7}\\ p_{6}⊕k_{1}\\ p_{7}⊕k_{8}\\ p_{8}⊕k_{6}\\ p_{9}⊕k_{2}\\ p_{10}⊕k_{12}\\ p_{11}⊕k_{4}\\ p_{12}⊕k_{13}\\ p_{13}⊕k_{15}\\ p_{14}⊕k_{0}\\ p_{15}⊕k_{11} \end{array}\right]}^{⊤}.$$

Note that the learned cryptosystems of Equations (24) are secure, namely, the OTP. In our tests, just one model was a failure as Alice and Bob could not communicate. Almost all successful models trained under the proposed CPA-ANC methodology learned an OTP cryptosystem. Moreover, comparing the results of Table 2 and Table 3, one can note an increased number of successful trials. A plausible reason is that the original ANC methodology leads to a weaker adversary, and because of that, Alice and Bob have too much degrees of freedom with many possible solutions. This may lead to a more complex objective function with many local solutions. With CPA-ANC methodology and with the neural networks used, the only hope for Alice and Bob is to find an OTP solution. Therefore, it is likely that the objective function has a better behavior than the one obtained with the original ANC model.

## 5. Comparison with Related Work

As aforementioned, several NN cryptosystems were broken. This work reconstructs the technique ANC showing the cryptosystems generated by it. In addition, some of these cryptosystems are indeed Vigenère like ciphers, which can be broken. Furthermore, we show that the learning process from previous work probably will not generate a secure cryptosystem. Moreover, we improved the learning process by means of a stronger adversary, i.e., CPA-ANC and the same NN became able to learn an unbreakable cryptosystem with high probability, namely the OTP. Certainly, a cryptosystem is secure under the OTP assumptions, namely the one-time keys should be truly random and never leaked. The *CryptoNet* learned how to use the key. Table 4 summarizes the comparison.

Normally, the cryptosystem result from ANC is a type of Vigenère cipher and from CPA-ANC is OTP. We do not need to compare their complexity like in [23], because both cryptosystems require only XOR and have equivalent performance.

Research in the intersection between Security and Artificial Intelligence has a lot of challenges. To clarify our contribution, this work presents the first Artificial Neural Network able to learn an unbreakable cryptographic technique, namely OTP. Even with the advent of quantum computers or any other computing technology [24], we cannot break the OTP. Machines just learned how to use it.

## 6. Conclusions

In this paper, we have shown that a neural network can learn a perfectly secure cryptosystem in the right circumstances. In addition, we have shown that the original ANC methodology is not good enough to achieve this goal. Moreover, we have presented a new CPA-ANC methodology capable of improving the objective function and the learned model.

In our experiments, we used simple neural networks to better understand the learned model. Using the original ANC methodology, very few of the learned models were truly secure. However, using the proposed CPA-ANC almost all the learned models were an OTP, which is secure. The main message here is that the adversary must be very powerful to force the solution into a strong cryptosystem. In other words, the proposed CPA-ANC methodology alone is not enough to guarantee security, the design of a very strong adversarial capable of breaking cryptosystems is key. In our minimalistic model, this was possible to achieve, however, in general, this is a hard open problem.

For further work, the research will continue to conduct more tests to evaluate more parameters than the current tests. It is necessary to implement a parallel code to increase performance and test the proposed model with larger keys. Additionally, it remains an open problem whether a neural network can learn a secure cryptosystem in which a small key is used to encrypt a very long message, like a block or stream cipher would do.

Of course, we do not recommend using neural networks in real systems. This paper shows that is very likely to get a weak encryption scheme using neural networks. There is a long path ahead to understand in which conditions a complex neural network will learn a secure cryptosystem consistently. Eventually, neural networks might break current cryptosystems and create others more secure. It is a long journey to transform this theoretical result to a practical one.

## Figures and Tables

**Table 1:** 10 networks were learned and tested using Algorithm 1 for each key size. All the trials were successful since Alice and Bob were able to communicate without errors. However, all the trials resulted in an insecure algorithm.

Size of Key	Number of Trials	Successful Communications	Secure Algorithm Learned (OTP)
4-bit	10	10	0
8-bit	10	10	0
16-bit	10	10	0

**Table 2:** 20 networks were learned with ANC and tested using Algorithm 1 for each key size. Not all trials were successful since Alice and Bob were not able to communicate without errors in some cases. The network learned a secure algorithm (OTP) in some rare cases.

Size of Key	Number of Trials	Successful Communications	Secure Algorithm Learned (OTP)
4-bit	20	20	2
8-bit	20	18	2
16-bit	20	11	0

**Table 3:** 20 networks were learned CPA-ANC and tested using Algorithm 1 for each key size. Most of the trials were successful since Alice and Bob were able to communicate without errors. Most of the successful networks learned the OTP, which is a secure cryptosystem.

Size of Key	Number of Trials	Successful Communications	Secure Algorithm Learned (OTP)
4-bit	20	19	19
8-bit	20	20	20
16-bit	20	20	19

**Table 4:** Comparison with related work.

Work	Technique	Conclusion
[6]	chaotic synchronization aa	Broken in [10]
[7]	chaotic NN	Broken in [11]
[8]	chaotic NN	Broken in [12]
[9]	chaotic NN	Broken in [13]
[14]	ANC	converge to OTP with low probability.
This work aa	CPA-ANC	converge to OTP with high probability.

## Abbreviations

The following abbreviations are used in this manuscript:

AI	Artificial Intelligence
ANC	Adversarial Neural Cryptography
OTP	One-Time Pad
CPA	Chosen-Plaintext Attack
NN	Neural Networks
PRNG	pseudo-random number generators
